# A Hospital-Based Study of Iodine Nutrition Status of Breastfeeding Mothers in Bangladesh

**DOI:** 10.1155/2019/9896159

**Published:** 2019-10-07

**Authors:** Jobaida Naznin, Mohammad Fariduddin, Mashfiqul Hasan, Mohammad Atiqur-Rahman, Nusrat Sultana, Mohammad Anowar-Hossain, Sharmin Chowdhury, Muhammad Abul Hasanat

**Affiliations:** ^1^Department of Endocrinology, Bangabandhu Sheikh Mujib Medical University (BSMMU), Dhaka, Bangladesh; ^2^National Institute of Neurosciences and Hospital (NINS & H), Dhaka, Bangladesh

## Abstract

Adequacy of iodine nutrition status in breastfeeding mothers is vital in preventing iodine deficiency disorder (IDD) in neonates and children. The aim of the study was to assess urinary iodine status in breastfeeding mothers attending Bangabandhu Sheikh Mujib Medical University (BSMMU) hospital in Bangladesh. In this cross-sectional study carried out from January 2014 to January 2015, urinary iodine (UI; *μ*gm/L) level of 266 mothers (age 26.6 ± 4.7 years (mean ± SD), exclusively breastfeeding: 132 and nonexclusively breastfeeding: 134), recruited on consecutive basis from BSMMU outdoor and indoor, were measured in spot urine following the wet digestion method. Median UI in the participants was 298.6 (interquartile range, IQR 206.6–454.9) *μ*gm/L and only 6.4% lactating mother had low UI (i.e. <100 *μ*gm/L). There was no difference of median UI in relation to exclusive or nonexclusive breast feeding, presence of goiter, parity, and age of breastfed baby (*p*=ns for all). But median UI was higher in older subjects (≥30 years vs. <30 years: 364.4 (228.4–529.9) vs. 283.7 (205.4–434.0); median (IQR) *p*=0.040)), with good socioeconomic condition (good vs. average or less: 328.2 (243.8–510.0) vs. 274.4 (200.0–433.3); median (IQR); *p*=0.020), and in those who are aware regarding the importance of iodine (aware vs. unaware: 316.6 (225.2–506.3) vs. 270.1 (196.0–407.2); median (IQR); *p*=0.018). The proportion of participants with UI < 100 *μ*gm/L was similar in all the groups. Logistic regressions to predict deficient UI status revealed none of the variables to be an independent predictor. This study indicates that deficient iodine nutrition status in Bangladeshi breastfeeding mothers is not frequent at present.

## 1. Introduction

Breastfeeding plays an important role in the maintenance of iodine nutrition status of breastfed babies. Neonates and infants are target population of major concern for iodine deficiency disorder. They are particularly sensitive to iodine deficiency because of the low iodine content of their thyroid glands and an extremely fast turnover of intrathyroidal iodine. Hence, the iodine nutrition status of the breastfeeding mother is a concern [1]. To date, limited attention has been paid to their iodine status despite its importance in the intellectual development of young infants especially up to the second year of life [2–4]. Iodine deficiency is the single most important preventable cause of brain damage on a worldwide basis [5]. Children living in areas affected by severe iodine deficiency may have an intelligence quotient (IQ) of up to 13.5 points below that of comparable communities in areas where there is no iodine deficiency [6]. Bangladesh has enacted national legislation mandating compulsory salt iodization since 1989 which has practically been activated since 1993. Since then, iodine deficiency (urinary iodine excretion <100 *μ*gm/L) has been reversed to a remarkable extent [7]. But it has been emphasized by worldwide surveys that iodine deficiency disorder (IDD) can return at any time even after elimination if program success is not sustained. Also IDD can and does occur in unlikely areas like large cities and coastal areas. So, the need for continued vigilance is underlined [5].

In lactating period, iodine is concentrated in the mammary gland for more excretion in breast milk than through urine. Because of the need to ensure that the infant gets enough iodine from breast milk to build reserves in the thyroid gland, it was recommended that lactating women should continue to consume 250 micrograms of iodine per day same as during pregnancy [5, 8].

Iodine status is the most immediate measure of whether the thyroid gland has adequate iodine to function normally and protect the individual from the manifestations of iodine deficiency. Assessment of thyroid size, measurement of urinary iodine, thyroid stimulating hormone (TSH), and thyroglobulin are methods of assessing IDD prevalence. Since most countries have now started implementing IDD control programs, a WHO/UNICEF/ ICCIDD joint committee has emphasized urinary iodine as the principal indicator of impact. This indicator is highly accepted, and casual urine specimens are easy to obtain.

Most of the iodine (more than 90%) absorbed by the body is eventually excreted in the urine. The concentration of iodine in urine is an excellent indicator of recent iodine intake. Urinary iodine can vary somewhat from day to day and even within a given day in individuals. However, studies have convincingly demonstrated that a profile of iodine concentration in morning or other casual urine specimens provide an adequate assessment of a population when a sufficient number of specimens are collected [9, 10]. As urinary iodine values from populations are usually not normally distributed, the median rather than the mean should be used as the measure of central tendency.

For lactating women and children <2 years of age, a median urinary iodine concentration of 100 *μ*g/l can be used to define adequate iodine intake, but no other categories of iodine intake are defined [5]. Optimum iodine nutrition in lactating women and their iodine requirements have been mostly calculated depending on the mean breast milk excretion and mean breast milk iodine concentration (BMIC). It is also considered to be a good biomarker of the iodine nutrition status of breastfed neonates [11]. But there is no consensus on what an adequate BMIC is, and WHO has not made a recommendation on this issue [12]. So, we assessed the urinary iodine of breastfeeding mothers attending Bangabandhu Sheikh Mujib Medical University (BSMMU) hospital in Bangladesh to find out their iodine nutrition status.

## 2. Materials and Methods

### 2.1. Study Design

This cross-sectional study encompassed 270 breastfeeding healthy mothers from January 2014 to January 2015 by purposive nonprobability sampling. Mothers were attendants of patients in outdoor and indoor of BSMMU, Dhaka, Bangladesh. Those having known thyroid disorders, chronic diseases or history of taking any iodine-containing drug were excluded.

### 2.2. Study Procedure

Each recruited mother was interviewed and examined by the investigators. Socioeconomic condition was judged by gross monthly income of the family. Awareness regarding iodine was assessed simply by asking whether the participants know anything regarding the need for iodine. Five milliliters of spot urine sample was collected from each subject in screw capped deiodinized plastic cups and was transferred to two tightly sealed deiodinized Eppendorfs. These were preserved under −20°C in a refrigerator to avoid unpleasant odor. One Eppendorf from each sample was kept in reserve for replicate testing and for external quality control. During processing for assay in the laboratory, 4 (four) urine samples were accidentally dropped and thus these subjects were excluded from the test. Therefore, the final number of subjects was 266.

### 2.3. Ethical Aspects

Written informed consent was taken from all patients after completely explaining procedure, purpose, risk, and utility of the study. Approval of the institutional review board was taken for the study.

### 2.4. Analytic Methods

Samples were defrosted completely before analysis. Meticulous attention was given to avoid contamination with iodine at all stages. UI was estimated using the wet digestion method of Dunn et al. with modification of Sandell and Kolthoff involving colorimetric estimation of the rate of discolouration of ceric ammonium sulphate as an inverse measure of the organic iodine present. Iodine status (as deficient or sufficient) was considered on the basis of urinary iodine cutoff at 100 *μ*gm/l, as recommended by WHO as well as ICCIDD [5, 13, 14].

### 2.5. Statistical Analysis

Data from the study were analyzed using IBM SPSS Statistics for Windows version 22.0 (IBM corp, Armonk, NY, USA). Results are presented as mean (±SD) or median (interquartile range, IQR) and frequencies with percentage as applicable. Median UI of different groups was compared by Mann–Whitney *U* test and proportion of low UI by Chi-square or Fisher's exact test. Iodine status was dichotomized as adequate and deficient using cutoff value to see the independent influence of other factors such as age, parity, socioeconomic status, lactation type, presence of goiter, awareness of iodine, and age of breastfed baby by multiple logistic regressions analysis. *p* ≤ 0.05 was considered statistically significant.

## 3. Results

The present study included 266 breastfeeding mothers irrespective of the age of their child (ranging from 0 to 24 months) in a consecutive manner of which 132 were breastfeeding exclusively and the rest nonexclusively. Mean age of the participants was 26.6 ± 4.7 years. Most of the mothers were housewives (72.2%) followed by service holders (20.3%), students (4.9%), and others (2.6%). Majority of them were residing in Dhaka (68.8%), and socioeconomically, near half of them were of average socioeconomic condition. Educational status was secondary, higher secondary, and graduate levels in around 20% of mothers, while primary level education was around 30% and masters level about 12%. Near cent percent mothers (98.5%) were taking packet salt, and more than 60% mothers were aware of the need for taking iodized salt. Around 6% mothers had a family history of thyroid disease. Most of the subjects did not show any thyroid enlargement. However, grade 1 and grade 2 goiter were present in 12.4% and 2.4%, respectively (Table 1).

Median UI in the participants was 298.6 (IQR 206.6–454.9) *μ*gm/L, and only 6.4% lactating mother had low UI, that is < 100 *μ*gm/L. When median UI of different groups were compared, it was observed that there is no difference in relation to exclusive or nonexclusive breastfeeding, presence of goiter, parity, and age of breastfed baby. But median UI was higher in older subjects (≥30 years vs. <30 years: 364.4 (228.4–529.9) vs. 283.7 (205.4–434.0); median (IQR) *p*=0.040)), with good socioeconomic condition (good vs. average or less: 328.2 (243.8–510.0) vs. 274.4 (200.0–433.3); median (IQR); *p*=0.020), and in those who are aware regarding the importance of iodine (aware vs. unaware: 316.6 (225.2–506.3) vs. 270.1 (196.0–407.2); median (IQR); *p*=0.018). The proportion of participants with deficient iodine nutrition status as evidenced by low UI < 100 *μ*gm/L was similar in all the groups (Table 2). Multiple logistic regressions to predict deficient urinary iodine status revealed none of the variables to be an independent predictor (Table 3).

## 4. Discussion

The present study aimed at assessing iodine nutrition status in breastfeeding mothers in a backdrop of established universal salt iodization program in the country. Holding the cutoff for deficiency at UI 100 *μ*gm/L, this study clearly observed that about 94% mothers of the 266 studied subjects were sufficient with iodine nutrition status without any significant difference in proportion of sufficiency between different groups on the basis of age, socioeconomic condition, breastfeeding status, presence of goiter, awareness about iodine, parity, and age of breastfed baby. None of the variables could independently predict the deficient iodine nutrition status. However, median UI was higher in subjects with good socioeconomic condition, age >30 years and awareness regarding iodine. This findings are in agreement with some pilot studies in this country with exclusive breastfeeding mothers [15], while others in abroad observed varying results in the lactating mothers irrespective of exclusiveness of feeding; some found deficiency of UI [16, 17] and others found sufficiency [18–21].

Iodine nutrition status as assessed by median UI did not differ significantly between the exclusive and nonexclusive breastfeeding mothers in the present study. Moreover, the observed values in the present study were far higher than cutoff figure for the deficient state. It was interesting to observe that median level of UI was higher in the good socioeconomic group than in those with socioeconomic status average or less, in group aware of salt iodization than in those of the unaware group, and in the older age group (≥30 years) than in those of younger age group (<30 years). It seemed that awareness for iodized salt intake is important to improve UI. Effects of age and socioeconomic status of the mother on UI is also in concordance with other authors [22]. Median values in all groups from different views showed the level of UI far higher than the cutoff for the state of iodine deficiency. Therefore, it can be explained that the universal iodization program is working well and hopefully been adopted by the people of the country. National survey in 2004-5 showed that the prevalence of iodine deficiency was 38.6% in women [7]. Breastfeeding mothers were not an individualized subgroup despite being a vulnerable group. Nevertheless, there is concern of iodine excess in the children of these breastfeeding mothers [23]. In this context, it could be mentioned that in 2004 Hasanat et al. observed the frequency of iodine deficient state very less among the subjects in whom they were studying autoimmunity which was heightened [24]. Likewise, it is observed that iodine supplementation, when in excess, may cause iodine-induced hyperthyroidism [25, 26]. However, functional status was not evaluated in the present study; therefore, if any functional aberration was there at all, that was beyond the scope for discrimination in this study.

There was no difference of UI between goitrous and nongoitrous mothers, and in both groups, median values of UI were much high. Therefore, the presence of goiter in these mothers is unlikely to be attributable to iodine deficient state. However, frequency of goiter was around 15% among the subjects. Unlike the present findings, some investigators observed a bit higher frequencies of goiter among lactating mothers [27]; but, they also followed that frequency of deficient iodine status was minimum among goitrous subjects which virtually complies with the present findings. In this group, goiter may be related to the persistence of the physiological enlargement of thyroid size in pregnancy, to autoimmunity or unidentified goitrogen. Family history for thyroid diseases was less frequent, most subjects were housewives, indwelling inside Dhaka, socioeconomically good or average and virtually all subjects taking packet salt. Limited numbers of studies are there who did not find any remarkable link of iodine status with the demographic profile [16, 28].

We observed that none of the variables had significant predictive power over the deficient state of iodine. Persistent supplementation of iodized salt is needed to sustain a maintenance level of iodine nutrition status in the breastfeeding mothers.

For convenience of sample collection, the study included healthy breastfeeding mothers visiting the hospital as attendants and not seeking medical care for themselves. The true reflection of the community needs random sampling from different parts of the country and as such the present study may not reflect the iodine nutrition status of breastfeeding mothers of the community. We used Sandell–Kolthoff reaction for estimating UI. Despite being reliable and feasible, this method has some loopholes. There may be interference by other ions and the extreme values of UI may erroneously produce similar reading [29].

## 5. Conclusions

The present study observed that deficient iodine nutrition is not frequent among breastfeeding mothers. Although age, socioeconomic condition, and awareness of iodine have a positive impact, none may be good predictors for assessment over iodine nutrition status of lactating mothers.

## Figures and Tables

**Table 1:** Characteristics of the studied subjects.

Variables	Frequency (%)
No. of subjects	266
Age (mean ± SD, yr)	26.6 ± 4.7
Age range (yr)	18–38
Exclusively breast feeding	132 (49.6)
Occupation	
Housewife	192 (72.2)
Service	54 (20.3)
Student	13 (4.9)
Others	7 (2.6)
Area of residence	
Inside Dhaka	183 (68.8)
Outside	83 (31.2)
Socioeconomic condition	
Poor	40 (15.0)
Average	122 (45.9)
Good	104 (39.1)
Educational status	
Primary	81 (30.5)
SSC	43 (16.2)
HSC	55 (20.7)
Graduate	56 (21.1)
Masters	31 (11.7)
Parity	
Unipara	130 (48.9)
Multipara	136 (51.1)
Type of salt intake	
Open	4 (1.5)
Packet	262 (98.5)
Aware about iodine	166 (62.4)
Positive family history of thyroid disease	17 (6.4)
Presence of goiter	
Grade 1	33 (12.4)
Grade 2	6 (2.3)

(Within parentheses are percentages over column total).

**Table 2:** Urinary iodine of studied mothers.

Groups of mothers	Median UI with IQR in *μ*gm/L	*p* ^*∗*^	UI < 100 *μ*gm/L	UI ≥ 100 *μ*gm/L	*p* ^*∗∗*^
All subjects (*n* = 266)	298.6 (206.6–454.9)	—	17 (6.4)	249 (93.6)	—
Age group					
<30 years (*n* = 185)	283.7 (205.4–434.0)	**0.040**	10 (5.4)	175 (94.6)	0.321
≥30 years (*n* = 81)	364.4 (228.4–529.9)		7 (8.6)	74 (91.4)	
Socioeconomic condition					
Average or less (*n* = 162)	274.4 (200.0–433.3)	**0.020**	12 (7.4)	150 (92.6)	0.398
Good (*n* = 104)	328.2 (243.8–510.0)		5 (4.8)	99 (95.2)	
Breast feeding status					
Exclusively (*n* = 132)	284.6 (196.0–441.5)	0.262	6 (4.5)	126 (95.5)	0.222
Nonexclusively (*n* = 134)	316.6 (226.2–479.9)		11 (8.2)	123 (91.8)	
Presence of goiter					
Goitrous (*n* = 39)	302.0 (205.5–448.5)	0.925	4 (10.7)	35 (89.7)	0.288
Nongoitrous (*n* = 227)	297.4 (206.5–445.8)		13 (5.7)	214 (94.3)	
Awareness about iodine					
Aware (*n* = 166)	316.6 (225.2–506.3)	**0.018**	8 (4.8)	158 (95.2)	0.177
Unaware (*n* = 100)	270.1 (196.0–407.2)		9 (9.0)	91 (91.0)	
Parity					
Unipara (*n* = 130)	285.8 (210.7–436.8)	0.271	5 (3.8)	125 (96.2)	0.097
Multipara (*n* = 136)	319.7 (205.5–503.6)		12 (8.8)	124 (91.2)	
Age of breast fed baby					
≤6 months (*n* = 138)	290.8 (197.2–290.8)	0.370	7 (5.1)	131 (94.9)	0.361
>6 months (*n* = 128)	310.3 (226.1–473.2)		10 (7.8)	118 (92.2)	

^*∗*^by Mann–Whitney *U* test ^*∗∗*^by χ2 test/Fisher's exact test. Within parentheses are percentages over row total if not mentioned otherwise. UI: urinary iodine; IQR: interquartile range.

**Table 3:** Multiple logistic regressions for predictors of low urinary iodine (**<100** *μ*gm/L).

Variables	*p*	OR	95% CI for OR
Age of the mother ≥30 years	0.249	4.18	0.37–47.45
Socioeconomic status average or less	0.527	1.46	0.45–4.70
Nonexclusive breast feeding	0.173	2.21	0.71–6.88
Multiparity	0.183	2.32	0.67–7.99
Goiter	0.219	1.97	0.67–5.76
Unawareness of iodine	0.496	2.30	0.21–25.27
Age of breastfed baby ≤6 months	0.284	1.85	0.60–5.66

OR: odds ratio, CI: confidence interval.

## Data Availability

The data used to support the findings of this study are available from the corresponding author upon request.
